# A Novel Wistar Rat Model of Obesity-Related Nonalcoholic Fatty Liver Disease Induced by Sucrose-Rich Diet

**DOI:** 10.1155/2016/9127076

**Published:** 2015-12-14

**Authors:** Maria Luíza R. P. Lima, Laura H. R. Leite, Carolina R. Gioda, Fabíola O. P. Leme, Claudia A. Couto, Cândido C. Coimbra, Virginia H. R. Leite, Teresa Cristina A. Ferrari

**Affiliations:** ^1^Departamento de Clínica Médica, Faculdade de Medicina, Universidade Federal de Minas Gerais, Avenida Professor Alfredo Balena 190, 30130-100 Belo Horizonte, MG, Brazil; ^2^Departamento de Fisiologia, Instituto de Ciências Biológicas, Universidade Federal de Juiz de Fora, 36036-900 Juiz de Fora, MG, Brazil; ^3^Instituto de Ciências Biológicas, Universidade Federal do Rio Grande, Carreiros, 96203-900 Rio Grande, RS, Brazil; ^4^Departamento de Veterinária Clínica e Cirúrgica, Escola de Veterinária, Universidade Federal de Minas Gerais, 31270-901 Belo Horizonte, MG, Brazil; ^5^Departamento de Fisiologia e Biofísica, Instituto de Ciências Biológicas, Universidade Federal de Minas Gerais, 31270-901 Belo Horizonte, MG, Brazil; ^6^Departamento de Anatomia Patológica e Medicina Legal, Faculdade de Medicina, Universidade Federal de Minas Gerais, 30130-100 Belo Horizonte, MG, Brazil

## Abstract

The pathogenesis of nonalcoholic fatty liver disease (NAFLD) is not fully understood, and experimental models are an alternative to study this issue. We investigated the effects of a simple carbohydrate-rich diet on the development of obesity-related NAFLD and the impact of physical training on the metabolic abnormalities associated with this disorder. Sixty Wistar rats were randomly separated into experimental and control groups, which were fed with sucrose-enriched (18% simple carbohydrates) and standard diet, respectively. At the end of each experimental period (5, 10, 20, and 30 weeks), 6 animals from each group were sacrificed for blood tests and liver histology and immunohistochemistry. From weeks 25 to 30, 6 animals from each group underwent physical training. The experimental group animals developed obesity and NAFLD, characterized histopathologically by steatosis and hepatocellular ballooning, clinically by increased thoracic circumference and body mass index associated with hyperleptinemia, and metabolically by hyperglycemia, hyperinsulinemia, hypertriglyceridemia, increased levels of very low-density lipoprotein- (VLDL-) cholesterol, depletion of the antioxidants liver enzymes superoxide dismutase and catalase, and increased hepatic levels of malondialdehyde, an oxidative stress marker. Rats that underwent physical training showed increased high-density lipoprotein- (HDL-) cholesterol levels. In conclusion, a sucrose-rich diet induced obesity, insulin resistance, oxidative stress, and NAFLD in rats.

## 1. Introduction

Over the last decades, obesity has become a global epidemic and an important public health problem in many countries [1]. This condition is largely due to excessive consumption of saturated fats and simple sugars [2, 3], which, associated with sedentarism, represent the modern lifestyle [4]. Obesity is recognized as a risk factor for many disorders including type-2 diabetes and nonalcoholic fatty liver disease (NAFLD). NAFLD encompasses a spectrum of increasingly severe clinicopathological conditions ranging from fatty liver to steatohepatitis (NASH) with or without hepatic fibrosis/cirrhosis. Recent evidence suggests that NAFLD is also associated with cardiovascular and chronic kidney disease [5] and increased risk of hepatocellular carcinoma [5–8].

It has been considered that insulin resistance and hyperinsulinemia play a key role in the pathogenesis of NALFD (first causative step). Excessive deposition of fat in adipocytes and muscles determines insulin resistance with subsequent accumulation of fat in the liver [9], which, in turn, increases the rate of mitochondrial beta-oxidation of fatty acids and ketogenesis that can promote lipid peroxidation and accumulation of reactive oxygen species (ROS) in the hepatocytes [10, 11]. These compounds generate a variety of cellular stimulations with subsequent inflammatory response, which has been recognized as the causal factor of NASH/fibrosis (second causative step) [12, 13].

In spite of growing knowledge, several aspects of NAFLD pathogenesis are still unknown. Considering the difficulty in developing human studies to evaluate the influence of nutrition in the development of NAFLD and associated metabolic abnormalities, experimental models constitute a reliable alternative way. Different animal models of NAFLD/NASH have been developed, but few of them replicate the entire human phenotype [12, 14]. These models may be classified into three basic categories: those caused by either spontaneous or induced genetic mutation; those produced by either dietary or pharmacological manipulation; and those involving genetic mutation and dietary or chemical challenges. The dietary manipulations used in these last two types of models usually do not resemble human dietary pattern. In the present study, we developed a model of obesity and obesity-related NAFLD in nongenetically modified Wistar rats using a simple carbohydrate-rich diet, which resembles the current dietary pattern of humans, and followed the sequence of the pathophysiologic events and their clinical and metabolic consequences. In this context, it should be noted that, in the vast majority of studies on NAFLD in which animal models were employed, the description of the sequence of the pathophysiologic events and their consequences have not been addressed, as their key goal is usually the evaluation of a specific aspect such as a therapeutic intervention. Furthermore, we evaluated the impact of physical training on the metabolic abnormalities associated with this disorder.

## 2. Material and Methods

### 2.1. Animals and Experimental Design

Sixty male Wistar rats, approximately 28 days old (after weaning), were housed individually and had free access to water and rat diet. The animals were randomly separated into the following groups: experimental group (EG), fed with highly palatable diet (see below) during 5 (EG5, 6 rats), 10 (EG10, 6 rats), 20 (EG20, 6 rats), and 30 (EG30, 12 rats) weeks, and control group (CG), fed with standard rat chow during 5 (CG5, 6 rats), 10 (CG10, 6 rats), 20 (CG20, 6 rats), and 30 (CG30, 12 rats) weeks. From week 25 to week 30, 12 animals belonging to the EG30 (6 rats) and CG30 (6 rats) were submitted to physical training (see below).

At the end of each experimental period, after fasting for 10 hours, the animals were sacrificed. Blood samples were taken by cardiac puncture and stored at −20°C. The livers were immediately removed and fragments of about 1 mm thickness were fixed in 4% formaldehyde, dehydrated, immersed in xylene, and then embedded in paraffin for histology. Fresh tissue samples were collected to evaluate antioxidant enzymes activity.

All experiments were approved by the Ethics Committee of the Universidade Federal de Minas Gerais for the Care and Use of Laboratory Animals (CETEA 53/2007) and were carried out in accordance with the regulations described in the Committee's Guiding Principles Manual. A rat belonging to the CG20 died and was excluded from all analyses.

### 2.2. Diet

The standard rat chow (Nuvilab-CR1 Nuvital-Colombo, Brazil) had the following nutrient composition: protein, 22%; fat, 4%; carbohydrate, 42%; minerals, 10%; phosphorus, 0.8%; vitamins, 1%; fiber, 8%; water, 12.5%. The chemical analysis revealed that 100 g of this diet contained 309 kcal, 24.8 g of protein, 3.4 g of fat, 44.8 g of carbohydrates, 8.2 g of fixed mineral residue, and 18.8 g of dietary fiber. The diet known as effective in inducing obesity in rats and described as highly palatable was composed of what follows: 33% of standard rat chow compacted to powder, 33% of condensed milk (Moça, Nestlé, Brazil), 7% of sucrose (refined sugar, União, Brazil), and 27% of water [15]. The condensed milk was nutritionally composed of carbohydrate, 56.7%; fat, 8.3%; protein, 6.7%; water, 28.3%. According to the chemical analysis, 100 g of dried highly palatable diet contained 339 kcal, 16.1 g of protein, 3.4 g of fat, 61 g of carbohydrates (18% of simple carbohydrates), 5.1 g of fixed mineral residue, and 14.4 g of dietary fiber.

The diet was prepared daily, weighed, fractionated in portions, and stored in the feeder for 8–10 hours. The remaining food in the feeder was weighed to calculate the final amount of ingested food. The water content of the drinking bottles was renewed daily.

### 2.3. Anthropometric Parameters and Physical Training

On a weekly basis, the body weight, thoracic circumference (TC) (measured between the foreleg and hind leg), and nasoanal length were measured. Body mass index (BMI), that is, the ratio between body weight (g) and the square of body length (cm²), was calculated [16].

All animals were acclimatized to exercise on the motor-driven treadmill (Gaustec, Brazil) by running at a speed of 10 m·min^−1^ at 5% inclination for 5 minutes/day, during 5 consecutive days. After exercise familiarization, trained rats were submitted to the physical training protocol, which consisted of running sessions with gradual increase in intensity across 5 weeks, 5 days/week. The speed and duration of the exercise bouts were increased until the rats were able to run at 25 m·min^−1^, 5% inclination, during 60 minutes/day. The achievement of this exercise intensity ensures that a significant endurance training effect is produced [17]. In order to ensure that all animals were subjected to the same handling stress, untrained group was submitted to running exercise on the same days of physical training, at the same speed, but for 2 minutes only [17].

### 2.4. Analytical Procedures of Blood Parameters

Measurement of glucose, total cholesterol, very low-density lipoprotein- (VLDL-) cholesterol, low-density lipoprotein (LDL-) cholesterol, high-density lipoprotein- (HDL-) cholesterol, and triglycerides was performed as recommended by the manufacturer (Bioclin, Quimbasa, Basic Chemistry Ltda, Brazil) using an autoanalyzer (StatPlus 2300, Yellow Spring Inst, USA).

Serum concentrations of leptin and insulin were determined by radioimmunoassay (Rat Leptin Ria Kit, Rat Insulin Ria Kit, LINCO Research, USA) using a gamma-ray counter (Mor-ABBOT, USA). The minimum detection value was 0.5 ng/mL.

### 2.5. Evaluation of Antioxidant Enzyme Activity

The determination of superoxide dismutase (SOD) activity was adapted from Dieterich et al. [18]. Briefly, fresh liver samples were homogenized in 50 mM sodium phosphate buffer (1 mL, pH 7.8, 37°C) and 1 mM of diethylenetriamine pentaacetic acid (DTPA), immediately after their removal. The reaction was initiated by addition of pyrogallol acid (0.2 mM/L, 37°C for 3 minutes) and the absorbance measured at 420 nm. SOD activity was calculated as U/mg protein, where 1 U of the enzyme was defined as the amount required to inhibit the oxidation of pyrogallol by 50%.

Catalase (CAT) activity was measured in the supernatant of liver homogenate as described by Nelson and Kiesow [19]. Briefly, 0.04 mL of H_2_O_2_, 0.06 mL of liver homogenate, and 1.9 mL of potassium phosphate buffer (50 mM, pH 7.0) were mixed to give a final concentration of 6 mM of H_2_O_2_. It took 1 minute for the reaction to occur at room temperature. The decomposition of H_2_O_2_ by CAT was evaluated by the change in absorbance at 240 nm. The experiments were performed in duplicate. CAT activity was expressed as mmol of H_2_O_2_ decomposed per minute per milligram of protein. This procedure was adopted to avoid the possibility of interference in the activity of glutathione peroxidase, once the necessary cofactors were not present in the reaction medium.

### 2.6. Histological and Immunohistochemistry Evaluations

Histological sections were prepared from the material embedded in paraffin and stained with hematoxylin-eosin. The histological analysis was performed simultaneously by two examiners. The criteria established by Brunt et al. were used to describe the histological lesions. According to these criteria, macrovesicular steatosis is quantified based on the percentage of involved hepatocytes (0 = absent; 1 < 33%; 2 = 33–66%; 3 > 66%), and its zonal distribution and the presence of microvesicular steatosis are noted; hepatocellular ballooning is evaluated for zonal location, and the estimate of its severity (mild, marked) is based on the numbers of hepatocytes showing this abnormality [20].

Hepatic expression of malondialdehyde (MDA), leptin, and the leptin receptor Ob-R was evaluated by immunohistochemistry in the animals sacrificed at weeks 20 and 30. From paraffin embedded tissues, sections on salinized slides (4 mm) were collected, deparaffinized, and hydrated. For immunohistochemistry, antigen reaction with ethylenediaminetetraacetic acid (EDTA) at pH 8.0, no steamer for 30 minutes at 98°C, was conducted, followed by Tris HCl pH 7.6 washing. The whole procedure was performed using Polymer Detection System kit (Novolink Polymer Detection System, Novocastra, USA). The primary antibodies used were anti-MDA monoclonal antibody (1F83) (Cosmo Bio Co., Ltd., Japan) diluted in 0.5 mL; anti-Ob (A-20) sc-84; and anti-Ob-R (H-300) sc-8325 (Santa Cruz Biotechnology Inc., USA) at a dilution of 1 : 250 and 1 : 100, respectively.

### 2.7. Statistical Analysis

Data are presented as frequencies and percentages, mean ± standard deviation (SD), and median and interquartile range (IQR). For each quantitative response's variables, we developed linear regression models in which all variables with *P* value ≤0.25 at univariate analysis would be included initially. However, due to the high level of correlation between the explanatory covariates, we opted to adjust the final model with the following covariates: group, physical training, variation in BMI (ΔBMI), and variation in the amount of ingested calories (ΔKcal). The adequacy of the models was assessed by analysis of the residues. For the categorical variables, logistic regression models were developed, with inclusion of the variables that showed on the univariate analysis a *P* value ≤0.25, and also clinical significance. The model fit was assessed by the Hosmer-Lemeshow test. Statistical analysis was performed using the R public domain software. Significance level was set at *P* value <0.05.

## 3. Results

### 3.1. Descriptive Analysis of the Variables and Comparison between EG and CG

**Figure 1:**
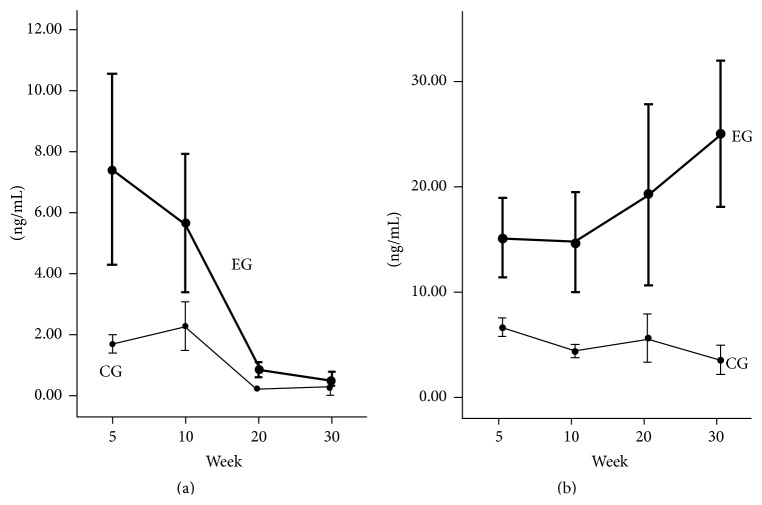
Levels of insulin and leptin over time. Mean and standard deviation of (a) insulin and (b) leptin serum concentrations, over time, in the experimental (EG) and control (CG) groups.

The results of the anthropometric parameters, lipid and glucose profile, hormones levels, and antioxidant enzymes activity, as well the results of their comparative analyses between EG and CG along the time of follow-up, are described in Tables 1, 2, and 3. Insulin (Figure 1(a)) and leptin (Figure 1(b)) serum levels varied inversely over time in the EG.

**Figure 2:**
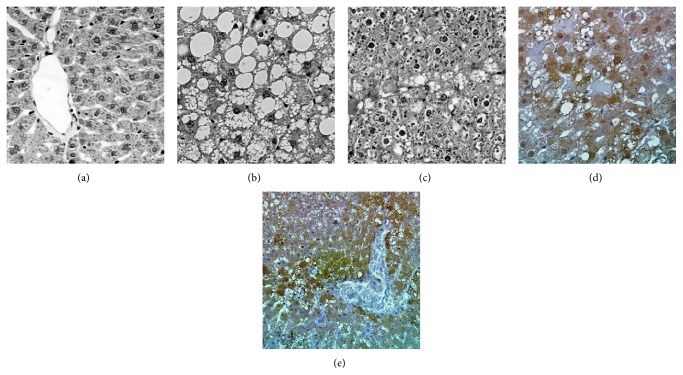
Liver histology and immunohistochemistry. (a) Rats fed with standard diet (control group) at 30 weeks; normal histology, hematoxylin and eosin stain ×10. (b, c, d, and e) Rats fed a sucrose-rich diet (experimental group) at 30 weeks; (b) macro and micro vacuolar steatosis, and hepatocellular ballooning, hematoxylin and eosin stain, ×40; (c) macro and micro vacuolar steatosis, and hepatocellular ballooning, hematoxylin and eosin stain, ×10. (d) Intense reaction to malondialdehyde ×40; (e) reaction to leptin ×20.

Liver histology was normal (Figure 2(a)) in the CG in all times of the experiment. Steatosis and hepatocellular ballooning (Figures 2(b) and 2(c)) were observed only in the EG, from week 10. Steatosis was macro- and microvacuolar, located predominantly in zone 3 of the liver acinus. The intensity of the macrovacuolar steatosis varied from mild (involvement of less than 33% of the hepatocytes) to severe (involvement of more than 66% of the hepatocytes) regardless of the time of the experiment. Ballooning was localized in zones 2 and 3 of the acinus, ranging from mild to marked and mismatched with the time of experiment. Neither inflammatory foci nor fibrosis was observed.

The reaction for identifying MDA (Figure 2(d)) was positive and intense, of cytoplasmic localization in zone 3 of the hepatic acinus, around the central vein, in EG20 and EG30. No MDA was detected in CG rats. Leptin (Figure 2(e)) was identified in the cytoplasm especially in zone 3 of the acinus, in EG20 and EG30. In CG rats, the reaction was weakly positive, at the same location. Ob-R was expressed as a weak cytoplasmic reaction predominantly in zone 3 of the acinus, in the rats of both groups, at weeks 20 and 30.

The comparison of the different variables between physical trained and untrained groups showed higher serum levels of HDL-cholesterol in the first group: medians 75 mg/dL and 52.2 mg/dL, respectively (*P* = 0.007). No other clinical or metabolic variable was significantly different between the groups after the physical training.

### 3.2. Multivariate Analysis

Table 4 shows the results of the final linear and logistic regressions models. In summary, blood glucose levels were 49% higher in EG rats than in CG rats, and the rats studied for 10 and 30 weeks had an increase of 49% and 65%, respectively, in serum glucose compared to those studied for 5 weeks. Total cholesterol was 19.2 mg/dL higher in the EG in comparison with the CG. Rats undergoing physical training showed an average of 27.1 mg/dL increase in HDL-cholesterol than those that did not exercise; and each increase of 1 unit in Δkcal intake caused an average reduction of 0.03 mg/dL in HDL-cholesterol levels. Regarding LDL-cholesterol, there was an average increase of 60.2 mg/dL for each increase of 1 unit in ΔBMI.

Two models were adjusted for the dependent variable insulin. The first, composed by the time (categorical) and groups of rats, showed that the EG20 and EG30 had, respectively, lower insulin values of 83% and 89% compared to EG5. Furthermore, the animals of EG had an average insulin levels increased by 123% compared to the CG. The second model, including time (quantitative form), groups of rats, and Δkcal intake, showed that, for each increase of 1 unit in time, the average value of insulin decreased by 7% and, for each increase of 1 unit in Δkcal intake, the average value of insulin increased by 0.2%. The EG rats had an average insulin level increased by 100% compared to those of the CG.

In EG20 and EG30, the leptin values were 33% and 40% higher, respectively, compared to the rats followed for 5 weeks. The EG had a mean value of leptin increased by 267% compared to the CG, and for every increase of 1 unit in ΔBMI the average value of leptin increased by 124.6%. The amount of SOD was 24% lower in the animals followed for 30 weeks in relation to those studied for 5 weeks. In the EG, the mean values of SOD were 11% lower compared to the CG; and, for each increase of 1 unit in ΔBMI, the mean SOD values decreased by 54%. The rats studied for 20 weeks presented an average of 2.4 less CAT units than those studied for 5 weeks, and in the EG an average of 1.8 less units of CAT relative to the CG was observed. Concerning the histological findings, it was found that, for each increase of 1 unit in the ΔTC, the chance of expressing ballooning and steatosis increased by 50%.

## 4. Discussion

This study demonstrates that a diet with high amount of simple carbohydrates, which resembles the current human dietary pattern, was able to induce obesity-related NAFLD, here characterized histologically by hepatic steatosis and hepatocyte ballooning, clinically by increased TC and BMI associated with hyperleptinemia, and metabolically by hyperglycemia, hyperinsulinemia (with subsequent insulin return to baseline levels), hypertriglyceridemia, increased serum levels of VLDL-cholesterol, depletion of antioxidants liver enzymes, and increased levels of MDA, an oxidative stress marker. Furthermore, rats that underwent physical training showed a significant increase in HDL-cholesterol in comparison to those that did not exercise.

High-fat and methionine choline-deficient diets are widely used to produce hepatic steatosis and NASH in experimental animals [12, 14, 21–26]. However, these diets do not reflect the usual dietary pattern of humans regarding their composition. Diets high in both saturated fat and simple carbohydrate have also been commonly used in genetically modified or wild-type animals in experimental models of NAFLD [27–35]. Animal models in which NAFLD was induced by simple carbohydrate-rich diets (usually fructose) are less numerous, and in most of them only hepatic steatosis was observed [28, 36–44]. Although the animal models that combine naturally occurring or induced genetic mutations associated with dietary or chemical challenges resemble the histopathology and pathophysiology of human NAFLD more closely, the dietary challenge is usually performed by high-fat or methionine choline-deficient diets [12, 14, 45–47]. Although each of these models is valuable, they fail to address key aspects of the process in humans. For example, few humans have diets that are deficient in methionine and choline. Moreover, rodents exposed to methionine- and choline-deficient diets are not obese; rather, they lose weight and become more insulin-sensitive [48]. On the other hand, the diet used in our investigation was balanced in terms of its content in proteins, lipids, carbohydrates, vitamins, and minerals, in addition to being highly palatable, normocaloric, and fiber containing. Furthermore, it was administered in solid consistency, as pellets, during a relatively long period of time. What has usually been described in the other animal investigations is a rapid induction of obesity due to the administration, in a short period of time, of a high-caloric high-fructose and/or high-fat diet, as liquid in troughs or via a nasogastric tube. In synthesis, we sought to feed the animals with a diet as similar as possible to a normal diet regarding its content as well as its form of administration.

In our study, free access to the sucrose-rich diet and high food consumption caused obesity/abdominal obesity in the EG rats from week 10. Obesity was associated with increased serum levels of glucose, triglycerides, VLDL-cholesterol, and insulin, which are manifestations of insulin resistance [9, 49]. The hyperinsulinemia led to increased hepatic synthesis of fatty acids, triglyceride accumulation in the hepatocytes, with subsequent steatosis. Surplus triglyceride was exported as VLDL-lipoprotein. The* de novo* hepatic lipogenesis, which is aggravated by diets with higher carbohydrate content than fat, plays an important role in glucose homeostasis and development of hypertriglyceridemia and hyperinsulinemia [50, 51]. For example, when the amount of ingested carbohydrate exceeds the total calorie needs, the rate of* de novo* hepatic lipogenesis increases by 10 times [52]. Likewise, this rate increases 27 times with the ingestion of a diet with high carbohydrate content compared to low-carbohydrate diets and fasting [53].

A positive correlation between increase in serum levels of leptin and BMI was another finding of this study that corroborates human observations [54]. The hyperleptinemia may be not only a consequence of hyperphagia and obesity, but also a result of the fructose component of the diet, which is in agreement with the study by Vilà et al., which demonstrated induction of hyperleptinemia by fructose [55]. In humans, increased levels of leptin are observed in obese individuals and in patients with NAFLD/NASH. It is suggested that this increase may reflect a state of leptin resistance at central level as well in the muscles and liver [56, 57].

In an attempt to understand the action of leptin in the liver and its possible role in the pathogenesis of NAFLD/NASH, we evaluated the expression of leptin and Ob-R in the hepatic parenchyma and found intense leptin reaction in EG30, whereas Ob-R was observed in both groups, without difference between them. A possible role of leptin as an inducer of hepatic mitochondrial beta-oxidation has been postulated. Huang et al. demonstrated that leptin* in vivo* enhances the activity of the fatty acid oxidative pathway in the liver, thus contributing to the reduction of triglycerides and VLDL-cholesterol in rats without leptin resistance [58]. On the other hand, some authors observed increased mitochondrial beta-oxidation in the liver of leptin deficient mice (ob/ob) with severe steatosis [59]. Cao et al. showed that leptin, in the long term, can cause hepatic fibrosis due to the increase of the local levels of oxidative stress [60]. Therefore, it is possible to hypothesize that leptin may play a protective role in the early stages of NAFLD; and, at later stages, it may contribute to the development of fibrosis. Further studies are necessary to clarify the biological function of leptin in the normal liver and its possible role in diet-induced NAFLD.

Hepatic steatosis and hepatocellular ballooning—early stages of NAFLD—were present in all liver samples of the EG from week 10. At the final stage of the investigation, although more exuberant steatosis was expected, the pattern was similar to that observed at week 10. The duration of the study may not have been long enough to allow the development of more severe steatosis and the histological changes that characterize NASH. As the hepatic lesions that occur in NASH are associated with the expression of proinflammatory cytokines in the liver, it is possible that their investigation could have demonstrated NASH at an early stage. In addition, genetic factors could be acting. It is also possible to speculate that the high levels of leptin could be exerting a protective effect.

MDA, a marker of lipid peroxidation, presented exuberant expression in the EG, whereas this reaction was negative in the CG. Oxidative stress induced by lipid peroxidation is a result of oxidant/antioxidant system imbalance [61]. Cellular stimulation by ROS and the subsequent inflammatory response have been described as the “second hit” that culminate with the development of NASH [62, 63]. In this context, we found in EG30 a reduction in the levels of the antioxidants enzymes SOD and CAT. This observation suggests that during the initial phases of the experiment there was a balance between antioxidants/prooxidants constituents; however, over time, an imbalance in favor of prooxidants was developed. The use of diets with high amounts of simple carbohydrates induces hypertriglyceridemia resulting in reduction of the antioxidants reserves [64, 65].

Although we observed hepatocellular ballooning denoting cell injury, one limitation of our study is the fact of not detecting NASH histologically. This was also a finding in several of the previous models in which NAFLD was induced by a simple carbohydrate-rich diet [28, 36–44]. As stated above, it is possible that the time of the experiment was not long enough to enable the development of the histological characteristics of NASH, which may require higher levels of ROS and/or longer exposure to the offending agent, in addition to liver susceptibility probably related to genetically determined factors, such as preexisting defects in mitochondrial oxidative phosphorylation [66, 67]. In the presence of intense and sustained production, ROS can cause damage to cell membranes, proteins, and DNA, leading to the release of proinflammatory cytokines, activation of hepatic stellate cells, fibrogenesis, and direct liver damage [68].

Exercise is considered an effective resource for controlling metabolic changes associated with obesity [69]. The physical training used in this study was effective in increasing HDL-cholesterol, corroborating the findings from a study in Zucker rats [70]. On the other hand, other authors found no significant effect on HDL-cholesterol in rats or mice submitted to physical training [71, 72]. No other metabolic parameter suffered alteration in response to physical exercise, which could have been due, at least partially, to the time not long enough of the physical training. In this context, 12 weeks of regular exercise reduced liver triglyceride content and serum levels of LDL-cholesterol in the KK/Ta mice fed a high-sucrose diet [72]. In humans, evidence suggests that regular exercise reduces the risk factors for NASH [1, 8].

## 5. Conclusion

Our study demonstrated that a diet enriched with sucrose induced obesity, insulin resistance, diabetes, oxidative stress, and subsequent hepatic steatosis and hepatocellular ballooning. The lack of histologically evident inflammation and fibrosis in the liver parenchyma may have been due to the insufficient time of the experiment.

## Figures and Tables

**Table 1:** Comparison of anthropometric parameters between experimental and control groups.

Time (weeks)	Groups		*P* value^*∗*^
	Experimental	Control	
ΔBMI (kg/cm^2^)^†^			
5	0.25 (±0.09)	0.36 (±0.07)	0.032
10	0.26 (±0.14)	0.27 (±0.08)	0.082
20	0.48 (±0.09)	0.40 (±0.10)	0.193
30	0.50 (±0.15)	0.34 (±0.06)	0.003

ΔThoracic circumference (cm)^†^			
5	6.67 (±0.45)	6.67 (±0.80)	1.000
10	10.48 (±1.86)	8.32 (±2.04)	0.083
20	12.00 (±1.27)	9.02 (±1.03)	0.002
30	13.82 (±2.84)	9.85 (±1.30)	0.031

^*∗*^ *t*-test; ^†^mean ± standard deviation.

**Table 2:** Comparison of biochemical parameters between experimental and control groups.

Time (weeks)	Groups		*P* value^*∗*^
	Experimental	Control	
Glucose (mg/dL)^†^			
5	272.3 (±92.5)	176.2 (±27.1)	0.035
10	434.5 (±214.5)	307.2 (±121.9)	0.235
20	324.2 (±53.4)	279.4 (±133.8)	0.468
30	463.1 (±101.7)	299.8 (±78.6)	<0.001

Total cholesterol (mg/dL)^†^			
5	83.5 (±18.0)	72.8 (±20.6)	0.363
10	64.5 (±13.1)	73.2 (±7.9)	0.194
20	108.0 (±33.3)	85.8 (±21.0)	0.231
30	104.3 (±33.6)	80.6 (±15.0)	0.099

HDL-cholesterol (mg/dL)^‡^			
5	49.1 (22.9–65.3)	39.2 (22.9–64.1)	0.779
10	34.8 (23.0–52.8)	42.3 (37.7–49.7)	0.689
20	49.6 (44.0–69.5)	57.9 (56.8–63.7)	0.315
30	65.8 (51.8–75.3)	64.0 (51.1–79.4)	1.000

VLDL-cholesterol (mg/dL)^‡^			
5	20.5 (14.3–25.9)	13.7 (10.9–18.9)	0.149
10	17.4 (14.1–35.7)	17.9 (13.5–19.2)	0.522
20	17.1 (11.8–24.3)	11.0 (9.0–14.1)	0.083
30	26.3 (22.1–49.8)	11.0 (9.1–17.0)	<0.001

LDL-cholesterol (mg/dL)^†^			
5	24.8 (±21.6)	24.8 (±12.2)	1.000
10	23.7 (±26.5)	12.7 (±6.0)	0.343
20	34.6 (±22.6)	19.5 (±16.6)	0.246
30	38.4 (±18.1)	15.0 (±10.7)	0.341

Triglycerides (mg/dL)^‡^			
5	102.5 (71.5–129.5)	68.5 (54.5–94.3)	0.150
10	87.0 (70.5–178.3)	89.5 (67.3–95.8)	0.522
20	85.5 (58.8–121.5)	55.0 (45.0–70.5)	0.083
30	131.5 (110.5–248.8)	55.0 (45.3–85.0)	<0.001

^*∗*^ *t*-test for normally distributed and Mann-Whitney test for nonnormally distributed variables; ^†^mean ± standard deviation; ^‡^median (interquartile range).

**Table 3:** Comparison of hormonal levels and enzyme activity between experimental and control groups.

Time (weeks)	Groups		*P* value^*∗*^
	Experimental	Control	
Insulin (*µ*L)^‡^			
5	7.6 (3.8–11.1)	1.6 (1.5–2.1)	0.005
10	4.7 (3.4–8.2)	1.7 (1.6–3.1)	0.013
20	0.9 (0.6–1.1)	0.3 (0.3–0.4)	0.008
30	0.4 (0.3–0.8)	0.2 (0.2–0.4)	0.043

Leptin (*µ*L)^‡^			
5	14.3 (11.7–18.9)	6.6 (5.8–7.6)	0.001
10	13.7 (9.6–20.9)	4.4 (3.6–4.8)	0.005
20	18.2 (10.3–25.9)	5.1 (3.3–8.1)	0.021
30	24.7 (13.1–37.0)	2.6 (1.8–5.7)	<0.001

Superoxide dismutase (U/mg protein)^†^			
5	1.5 (±0.1)	1.6 (±0.03)	0.090
10	1.6 (±0.02)	1.6 (±0.1)	1.000
20	1.6 (±0.1)	1.5 (±0.1)	0.288
30	0.9 (±0.3)	1.4 (±0.1)	<0.001

Catalase			
(mmol of H_2_O_2_ decomposed/minute/milligram of protein)^†^			
5	15.8 (±2.4)	15.9 (±2.7)	0.948
10	14.3 (±3.1)	15.9 (±2.7)	0.411
20	11.8 (±1.4)	15.1 (±2.9)	0.037
30	14.6 (±1.9)	16.7 (±3.5)	0.111

^*∗*^ *t*-test for normally distributed and Mann-Whitney test for nonnormally distributed variables; ^†^mean ± standard deviation; ^‡^median (interquartile range).

**Table 4:** Linear and logistic regression models for the response variables.

Variable/model	Coefficient (95% CI)	Coefficient exponential (95% CI)	*P *value
Glucose			
Constant	5.2		<0.001
Time (weeks)			
5			
10	0.4	1.49 (1.1; 2.0)	0.005
20	0.3	1.35 (1.0; 1.8)	0.090
30	0.5	1.65 (1.3; 2.1)	<0.001
Group			
EG	0.4	1.49 (1.3; 1.8)	<0.001
CG			

Total cholesterol			
Constant	177.7 (139.3; 216.1)		<0.001
Time (weeks)			
5			
10	−25.2 (−46.8; 3.6)		0.026
20	−50.9 (−107.7; 5.9)		0.085
30	−40.2 (−84.8; 4.4)		0.083
Group			
EG	19.2 (4.4; 31.2)		0.003
CG			
ΔKcal	−0.1 (−0.2; −0.03)		0.014

HDL-cholesterol			
Constant	53.1 (46.7; 59.5)		<0.001
Exercise			
Yes	26.1 (12.9; 39.4)		<0.001
No			
ΔKcal	−0.03 (−0.05; −0.01)		0.011

LDL-cholesterol			
Constant	2.8 (−16.3; 21.9)		0.774
ΔBMI	60.2 (11.9; 108.6)		0.018

Insulin (first model)			
Constant	0.8		<0.001
Time (weeks)			
5			
10	−0.005	1.00 (0.67; 1.47)	0.980
20	−1.8	0.17 (0.11; 0.24)	<0.001
30	−2.2	0.11 (0.07; 0.16)	<0.001
Group			
EG	0.8	2.23 (0.18; 2.70)	<0.001
CG			

Insulin (second model)			
Constant	0.7		0.011
Time (quantitative)	−0.07	0.93 (0.91; 0.95)	<0.001
Group			
EG	0.7	2.01 (1.65; 2.45)	<0.001
CG			
ΔKcal	0.002	1.002 (1.001; 1.003)	0.004

Leptin			
Constant	0.8		<0.001
Time (weeks)			
5			
10	−0.1	0.90 (0.61; 1.33)	0.450
20	−0.4	0.67 (0.45; 0.99)	0.047
30	−0.5	0.60 (0.41; 0.90)	0.004
Group			
EG	1.3	3.67 (3.01; 4.46)	<0.001
CG			
ΔBMI	2.6	13.46 (5.1; 35.87)	<0.001

Superoxide dismutase			
Constant	0.7		<0.001
Time (weeks)			
5			
10	−0.03	0.97 (0.85; 1.11)	0.606
20	0.10	1.11 (0.96; 1.27)	0.156
30	−0.27	0.76 (0.68; 0.86)	<0.001
Group			
EG	−0.12	0.89 (0.82; 0.96)	0.006
CG			
ΔIMC	−0.77	0.46 (0.32; 0.67)	<0.001

Catalase			
Constant	16.6 (15.0; 18.3)		<0.001
Time (weeks)			
5			
10	−0.7 (−2.9; 1.6)		0.564
20	−2.4 (−4.6; 0.2)		0.041
30	−0.1 (−2.1; 1.8)		0.902
Group			
EG	−1.8 (−3.2; 0.4)		0.016
CG			

Model/variable		Odds ratio (95% CI)	*P* value

Steatosis			
ΔTC		1.50 (1.10; 1.90)	0.002

Hepatocellular ballooning			
ΔTC		1.50 (1.10; 1.90)	0.002

EG, experimental group; CG, control group; Kcal, amount of calorie intake; TC, thoracic circumference.
